# Chicago Medical Society

**Published:** 1885-04

**Authors:** 


### LOCAL NEWS.

---

CHICAGO MEDICAL SOCIETY.—The thirty-fifth annual business meeting of the Chicago Medical Society was held on Monday evening, April 6th, 1885. The attendance was unusually large and representative.

The following officers, for the ensuing year, were elected by ballot:

*President.*—Professor C. T. Parkes, M. D.

*First Vice-President.*—C. W. Purdy, M. D.

*Second Vice-President.*—Professor James H. Etheridge, M. D.

*Treasurer.*—Harold N. Moyer, M. D.

*Secretary.*—Liston H. Montgomery, M. D.

*Members of the Committee on Membership and Miscellaneous Business.*—Gerhard C. Paoli, M. D.; Professor E. Fletcher Ingals, M. D.; Professor D. A. K. Steele, M. D.

*Member of the Committee on Library.*—F. C. Hotz, M. D.

Twenty-seven delegates to the annual meeting of the American Medical Association, at New Orleans, were elected.

The retiring President, Professor D. A. K. Steele, M. D., after a brief address,—in which he recommended the incorporation of the Society,—introduced the President-elect, Professor Charles T. Parkes, M. D.

The President-elect, Professor Charles T. Parkes, M. D., after a few appropriate remarks, called the Society to order.

As very general dissatisfaction was expressed with the manner in which the transactions of the Society had been reported during the past two years, the motion was carried, that a Committee on Publication, with power to investigate, should be appointed by the chair, the said committee to report at the next regular meeting of the Society.

The President appointed the following gentlemen: Professor David W. Graham, M. D., Edmund J. Doering, M. D., Robert Randolph, M, D.

Reports of committees were read, and important business transacted, before adjournment. The Transactions will appear in detail in the next issue of the CHICAGO MEDICAL JOURNAL AND EXAMINER.

